# Remission of primary hyperparathyroidism after fine needle aspiration of a parathyroid nodule mistaken for a thyroid nodule

**DOI:** 10.20945/2359-3997000000615

**Published:** 2023-06-19

**Authors:** Eugénia Maria Morado da Silva, Rute Alexandra da Costa Ferreira, Bernardo de Almeida Rodrigues Marques, Martinha Carlota Soares Chorão, João Manuel Sequeira Duarte

**Affiliations:** 1 Hospital de Egas Moniz Departamento de Endocrinologia Lisboa Portugal Departamento de Endocrinologia, Hospital de Egas Moniz, Lisboa, Portugal; 2 Hospital de Egas Moniz Departamento de Pathologia Lisboa Portugal Departamento de Pathologia, Hospital de Egas Moniz, Lisboa, Portugal

## Abstract

Primary hyperparathyroidism (PHPT) is an endocrine disorder characterized by hypercalcaemia and elevated or inappropriately normal concentrations of parathyroid hormone. Remission of PHPT caused by infarction or hemorrhage of a parathyroid adenoma rarely occurs, either spontaneously or induced, not always leading to a definitive cure. We report a case of 72-year-old women with primary hyperparathyroidism who underwent fine-needle aspiration cytology (FNAC) of a parathyroid adenoma mistaken for a thyroid nodule followed by normalization of parathyroid hormone (PTH) and serum calcium levels. Parathyroid origin was confirmed by immunohistochemistry. PTH levels began to rise at 4 months after FNAC demonstrating recurrence of the PHPT. This report shows that FNAC induced hemorrhage may cause remission of PHPT. Nevertheless, patient´s levels of PTH and serum calcium should be monitored, as remission may only be transitory.

## INTRODUCTION

Primary hyperparathyroidism is an endocrine disorder characterized by hypercalcemia and elevated or inappropriately normal concentrations of parathyroid hormone, caused primarily by a solitary parathyroid adenoma (80%-85%). The remaining 15%-20% of cases are associated with diffuse glandular hyperplasia, multiple adenomas, functional cysts, and parathyroid carcinoma (1). Surgery is usually the only definitive cure. Although rare, remission of primary hyperparathyroidism caused by infarction or hemorrhage of the parathyroid adenoma can occur, either spontaneously or induced, not always leading to a definitive cure (2). We report a case of a 72-year-old woman with primary hyperparathyroidism who underwent a fine needle aspiration biopsy (FNAB) of a parathyroid adenoma mistaken for a thyroid nodule. Within the following eight months, PTH levels decreased, and calcium serum levels normalized.

## CASE REPORT

**Figure 1:**
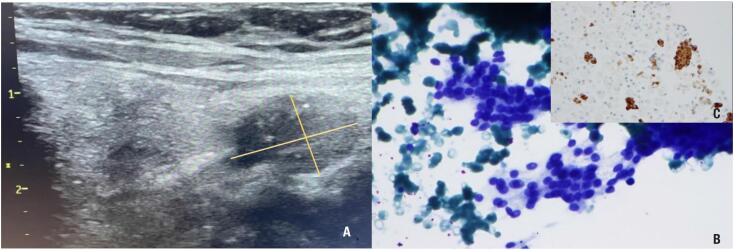
(**A**) Solid and hypoechoic nodule located next to the inferior pole of the left thyroid lobe in the US. (**B**) Parathyroid cytology showing the typical evenly stained round nuclei (Giemsa, 600x). (**C**) Positive parathyroid hormone immunostaining (PTH, 400x).

A 72-year-old woman with arterial hypertension, dyslipidemia, and osteopenia, was admitted to the emergency department (ED) due to persistent asthenia, obstipation, and polydipsia with two weeks of evolution. She was taking calcium carbonate and cholecalciferol 1.500 mg/400 UI twice daily, candesartan and hydrochlorothiazide 8+12,5 mg once daily and pitavastatin 2 mg once daily. There was no family history of hypercalcemia or urolithiasis. Biochemically, she had PTH-dependent hypercalcemia (Table 1) and an abdominal computed tomography (CT) showed a paralytic ileus. At observation no cervical mass was palpable. She was admitted and promptly initiated vigorous hydration and pamidronate intravenous infusion. The patient was discharged with a serum calcium of 12.4 mg/dL, and it was recommended to maintain vigorous hydration and to stop calcium carbonate, cholecalciferol, and hydrochlorothiazide. She was referred to an Internal Medicine appointment for further investigation: neck ultrasonography (US) found a predominantly cystic nodule with 40 mm in the inferior pole of the left thyroid lobe; the 99mTc-MIBI showed no uptake, either at the cervical or at the mediastinal level; the renal US did not show microlithiasis or microcalcifications; and bone mineral density was compatible with osteopenia (lumbar spine T-score -1.7 and femoral neck T-score -2.1). Meanwhile, the patient kept serum calcium values between 11.5 and 12.4 mg/dL. FNAB of the left thyroid nodule was performed and revealed a cystic hyperplasic nodule. Shortly after the FNAB, the patient showed neck pain and hematoma that resolved spontaneously. Laboratory evaluation two months after FNAB showed normalization of serum calcium and PTH levels (Table 2). Three months after FNAB she was sent to an endocrinology appointment due to an adrenal incidentaloma (classified as non-functioning adenoma after investigation). A thorough review of her medical history was done, and a neck US was performed due to the apparent remission of primary hyperparathyroidism. It revealed a 17.3 mm solid nodule next to the inferior pole of the left thyroid lobe, hypoechoic, with macrocalcifications (Figure 1A). Material obtained from the previously performed FNAB was reviewed at our institution and an immunohistochemistry study showed positive staining for PTH (Figures 1B and 1C), supporting the diagnosis of a parathyroid nodule. Eight months after FNAB, the patient remains under active surveillance, asymptomatic, with normocalcemia and mild elevated PTH (Table 2).

**Table 1:** Laboratory findings at emergency department and admission

Laboratory Parameter	Patient´s results at admission	Patient´s results after therapeutic	Reference Range
Albumin-corrected serum calcium (mg/dL)*	15.6	12.4	8.9-10.2
Phosphate (mg/dL)	1.9	2.2	2.5-4.5
Parathyroid hormone (pg/mL)	229.0	–	15.0-65.0
Magnesium (mEq/L)	1.7	1.7	1.6 – 2.4
25-hydroxyvitamin D (mmol/L)	98.0	–	75.0-250.0
Creatinine (mg/dL)	0.6	0.6	0.5 – 0.9
Glomerular Filtration Rate (ml/min/1.73m^2^)**	94.0	96.0	>60.0
Urinary Calcium (mg/24h)	–	180.0	100.0-300.0

* Corrected calcemia (mg/dL) = total serum calcemia (mg/dL) +0.8× (4 – serum albumin (g/dL)).

** Glomerular filtration rate (GFR) was calculated using the CKD-EPI formula.

**Table 2:** Laboratory findings after FNAB

Laboratory Parameter	Patient’s Results 2 months after FNAB	Patient’s Results 8 months after FNAB	Reference Range
Albumin-corrected serum calcium (mg/dL)*	9.9	9.4	8.9-10.2
Phosphate (mg/dL)	2.6	2.6	2.5-4.5
Parathyroid hormone (pg/mL)	43.2	71.8	15.0-65.0
Magnesium (mEq/L)	–	2.1	1.6-2.4
25-hydroxyvitamin D (mmol/L)	84.0	76.0	75.0-250.0
Creatinine (mg/dL)	0.6	0.6	0.50-0.90
GFR (mL/min/1.73 m^2^)**	95.0	94.0	>60.0
Urinary Calcium (mg/24 h)	–	214.0	100.0-300.0

* Corrected calcemia (mg/dL) = total serum calcemia (mg/dL) +0.8× (4 – serum albumin [g/dL]).

** Glomerular filtration rate (GFR) was calculated using the CKD-EPI formula.

## DISCUSSION

We report a rare case of a transient biochemical remission of primary hyperparathyroidism (PHPT) after FNAB was performed on a hyperfunctioning enlarged parathyroid lesion mistaken for a thyroid nodule. From our evaluation, the only cervical nodule was located next to the inferior pole of the left thyroid lobe and there were no thyroid nodules. This was not in agreement with the previous ultrasound. The ultrasound is an operator-dependent exam, and this nodule could easily be mistaken for a thyroid nodule or an intrathyroidal parathyroid adenoma (incidence of 1% to 6%). However, in this case, from our assessment, the nodule was clearly below the left thyroid lobe. The reduction in size could be explained by the fact that it was submitted to a FNAB and some cystic portions were aspirated. Normalization of serum calcium and PTH levels, along with the complaints of neck hematoma and pain, suggests that the FNAB may have induced a hemorrhage leading to remission of PHPT. PTH levels began to rise 4 months after FNAB, demonstrating recurrence of the PHPT. In literature, only four similar cases have been described, with the mechanism remaining unclear, although hemorrhage or infarction of a parathyroid lesion have been suggested as possible causes. In 2008, Ing and Pelliteri (3) reported a case of remission of PTHP after FNAB of an intrathyroidal parathyroid cyst which led to normocalcemia for at least 16 months of follow-up although PTH levels remained elevated. The authors suggested that the complete aspiration of the parathyroid cyst led to the normalization of serum calcium levels since there were no signs of hemorrhage. Maxwell and cols. (4) presented a case of remission of PHTP induced by FNAB that recurred 2 weeks later with rising levels of PTH. The authors suggested that the short time to recurrence was due to temporary ischemia and revascularization of the adenoma. Kara and cols. (2) described the longest follow-up of an FNAB-induced remission of PHPT, both biochemical and imagological, taking at least 9 years. Falcetta and cols. (5) reported a case of PHPT remission following FNAB with the patient presenting normocalcemia two weeks after FNAB and remaining in biochemical remission for at least 6 months. Based on these cases, patients´ levels of PTH and serum calcium should be monitored, as remission of PHPT following FNAB may be transitory, possibly due to regrowth after incomplete damage of the cellular component (2).

We must highlight the fact that FNAB is not recommended when PHPT is suspected but it can be useful when the location seems unusual to make sure the diagnosis is correct. FNAB can induce hemorrhage and can cause iatrogenic airway damage, hoarseness, or dysphagia due to compression (6). This procedure can also induce histological changes that can mimic malignant parathyroid lesions (7) and, rarely, can lead to parathyroid tissue implantation known as parathyromatosis (8). Therefore, it should not be done when parathyroid malignancy disease is suspected. In this case, parathyroid carcinoma was deemed unlikely because calcium levels after stopping her usual therapy (calcium carbonate, cholecalciferol, and hydrochlorothiazide) and PTH levels weren’t extremely high (serum calcium below 12.5 mg/dL and initial PTH of 229 pg/mL).

The differential diagnosis between parathyroid and thyroid lesions is difficult because of their similar location and cellular morphology. As in this case, the presence of follicular structures, oxyphilic cells, macrophages, and colloid-like material in smears may appear in both lesions and lead to misinterpretation of a parathyroid lesion for a thyroid lesion. Other features are more suggestive of a parathyroid lesion, such as the presence of a stippled chromatin pattern of nuclei, papillary-like clusters with vascular cores and clinging epithelial cells, and frequent occurrence of naked nuclei (9). Immunohistochemistry helps differentiate thyroid and parathyroid lesions and should be performed for an accurate diagnosis.

In the future, there is a need to try to understand the mechanisms behind this described phenomenon of induced-FNAB remission of PHPT and assess the possibility of less invasive therapies like percutaneous ethanol ablation, ultrasound-guided high-intensity focused ultrasound, or laser ablation. Although they should not be recommended as a primary therapy, these techniques may be useful, especially for patients who refuse or have contraindications for surgery.
